# Standardized Protocol for Resazurin-Based Viability Assays on A549 Cell Line for Improving Cytotoxicity Data Reliability

**DOI:** 10.3390/cells13231959

**Published:** 2024-11-26

**Authors:** Jessica Petiti, Sabrina Caria, Laura Revel, Mattia Pegoraro, Carla Divieto

**Affiliations:** 1 Division of Advanced Materials Metrology and Life Sciences, Istituto Nazionale di Ricerca Metrologica (INRIM), 10135 Turin, Italy; sabrina.caria@unito.it (S.C.); l.revel@inrim.it (L.R.); m.pegoraro@inrim.it (M.P.); c.divieto@inrim.it (C.D.); 2 Department of Chemistry, University of Turin, 10125 Turin, Italy

**Keywords:** cell viability assays, resazurin assay, A549 cell line, consistency of results, pre-clinical drug tests

## Abstract

The A549 cell line has become a cornerstone in biomedical research, particularly in cancer studies and serves as a critical tool in cytotoxicity studies and drug screening where it is used to evaluate the impact of pharmaceutical compounds on cellular viability. One of the most widely adopted methods for viability assessment, which is also used in evaluating drug cytotoxicity, is the resazurin-based assay. This assay exploits the ability of living cells to convert resazurin into fluorescent resorufin, providing a reliable indicator of metabolic activity. By measuring this conversion, cell viability can be estimated. Resazurin assay is extensively used for evaluating cytotoxic effects on various cell lines, including A549 cells, thereby bridging the gap between in vitro experimentation and drug development. However, frequent data inconsistencies in pre-clinical drug screening highlight the critical need for standardization to ensure reliability and reproducibility. This manuscript addresses these challenges by describing the optimization of resazurin-based viability assays for A549 cells in both 2D cultures and 3D fibrin gel models. By optimizing this test, the study aims to enhance the reliability of cytotoxicity results and introduces a new standard operating procedure, thus providing consistent results with minimal measurement uncertainty. This standardization is crucial for advancing drug screening and ensuring robust research findings.

## 1. Introduction

The A549 cell line, derived from the lung tissue of a 58-year-old white male with lung cancer, has emerged as a cornerstone in biomedical research, particularly in respiratory and cancer studies [1]. Characterized by epithelial morphology and adenocarcinoma origin, these cells show unique properties that make them well-suited for investigations into cell biology, drug screening, and toxicity testing. Over the years, A549 cells have significantly advanced the understanding of lung cancer and respiratory physiology, contributing to the overall progress of biomedical science. Furthermore, A549 cells have played a crucial role in scientific investigations concerning viral and bacterial infections. Indeed, researchers have utilized them to delve into the complexities of infectious diseases, including tuberculosis [2], since they serve as a model for studying the interaction between pathogens and host cells, shedding light on the molecular mechanisms that underlie infection progression [3]. Additionally, A549 cells have proven instrumental in the production of Adenovirus and contributed to advancements in gene therapy and viral vector research [4]. Their versatility extends beyond cancer studies, making A549 cells a key tool in several fields of biomedical research.

Among the most important applications of A549 cells, cytotoxicity studies, and drug screening are of particular relevance. Indeed, they play a pivotal role in evaluating the impact of pharmaceutical compounds on cellular viability. The sensitivity of A549 cells to different agents is particularly valuable for screening and profiling the cytotoxicity of novel pharmaceutical candidates. Moreover, their responsiveness to a broad range of chemical stimuli enables the exploration of drug interactions and mechanisms of action, thus establishing the A549 cell line as an indispensable tool in the early phases of drug discovery, which contributes to the development of safer and more effective therapeutic drugs [5].

The resazurin-based viability assay is one of the most adopted tools to assess drug cytotoxicity [6]. Exploiting the ability of living cells to reduce the non-fluorescent dye resazurin into fluorescent resorufin, this assay provides a reliable indicator of cellular metabolic activity and, consequently, cell viability [7]. The simplicity and sensitivity of the resazurin assay make it particularly advantageous for assessing the cytotoxic effects of various substances on several cell lines, including A549 cells [8]. Its versatility extends to high-throughput screening applications, enabling the rapid evaluation of several compounds and concentrations. The resazurin-based viability assay serves as a bridge between in vitro experimentation and drug development [9]. Indeed, it plays a pivotal role in the early stages of drug discovery, contributing to the identification of safe and effective compounds for further development and clinical exploration.

In this scenario, results consistency poses a significant challenge in pre-clinical drug screening. In fact, several papers identified data inconsistencies in pre-clinical studies [10,11]. In 2016, a Nature survey involving 1576 researchers highlighted that over 70% of the participants failed to replicate experiments conducted by other scientists. Additionally, more than 50% of researchers were unsuccessful in reproducing their own experiments [12]. Recently, two pharmacogenomic studies on over 450 cancer cell lines revealed significant discordance of data [13,14]. This inconsistency hampers result comparisons and raises concerns about the reliability of the findings. The choice of cell lines, such as the commonly used A549 cell line, introduces variability that can impact the repeatability of results. Experimental protocols, from reagent concentrations to assay conditions, must be standardized to mitigate inconsistencies. Additionally, biological complexity, such as cell culture conditions and health, further adds to the challenge. To address these issues, the scientific community is increasingly emphasizing transparent reporting, adherence to standardized methodologies, and collaboration for improving the robustness of pre-clinical research [15,16].

This manuscript outlines the optimization of resazurin-based viability assays on the A549 cell line in both 2D and 3D (fibrin gel) culture models, which utilizes and validates a standardized operating procedure (SOP) we previously developed [17], with the primary goal of enhancing the reliability of cytotoxicity data. As an outcome of this work, we provide a specific SOP for conducting resazurin-based cytotoxicity assays on A549 cells, which ensures reliable results with minimal uncertainty.

## 2. Materials and Methods

### 2.1. Cell Culture Conditions (2D)

The A549 cell line (CCL-185) was purchased from the American Type Culture Collection (ATCC). Cells were cultured in DMEM medium containing 1 g/L glucose, sodium pyruvate, and without L-Glutamine (Biowest, Nuaillé, France). The culture medium was supplemented with 10% heat-inactivated fetal bovine serum (FBS) (Sigma-Aldrich, St. Louis, MO, USA), 2 mM Glutamine (Lonza, Basilea, Switzerland), and 1% penicillin/streptomycin (Sigma-Aldrich, St. Louis, MO, USA). The cells were maintained at 37 °C with 5% CO_2_.

### 2.2. Resazurin Working Solution Preparation

Resazurin sodium salt (Invitrogen, Thermo Fisher Scientific, Waltham, MA, USA) was dissolved in 1× PBS (#A9162.0100, VWR, Radnor, PA, USA) to achieve a final concentration of 10 mM and stored at −20 °C. Intermediate dilutions (440 µM in 1× PBS) were prepared, sterilized using a 0.22 µm filter, and stored at −20 °C. Resazurin Working Solution (WS) was freshly prepared before each experiment by diluting 440 µM resazurin in complete DMEM, resulting in a final concentration of 44 µM.

### 2.3. Identification of Optimal Excitation and Emission Wavelengths

To optimize the assay, different combinations of excitation (Ex) and emission (Em) wavelengths (*λ*) were evaluated. A549 cells were detached following manufacturers’ recommendations and counted with a Neubauer chamber. Three different cell confluences were selected: ~9 × 10^4^ cells/cm^2^ for high confluency, ~4.5 × 10^4^ cells/cm^2^ for medium confluency, and ~9 × 10^3^ cells/cm^2^ for low confluency. Cells dilutions were appropriately prepared in complete DMEM (1% FBS) and seeded in triplicate in a 96-well plate (#655 180, Greiner Bio-One, Milan, Italy). Following an overnight (ON) incubation under standard culture conditions to ensure a firm attachment to the plate, the medium was gently removed, and 100 µL of resazurin WS was added to each well. Triplicate of resazurin WS only (Blank) was prepared for each *λ*_Ex_-*λ*_Em_ combination evaluated. After a 1.5 h (h) incubation, metabolized resazurin WS was transferred to a 96-well plate for fluorescence intensity (FI) detection (#MSSBNFX40, Millipore, Burlington, MA, USA). FI was measured using the EnSpire Multimode Plate Reader (Perkin Elmer, Waltham, MA, USA) at 12 *λ*_Ex_-*λ*_Em_ combinations, joining 4 *λ*_Ex_ (530, 535, 540, and 545 nm) with 3 *λ*_Em_ (585, 590, and 595 nm) [17].

### 2.4. Evaluation of Optimal Incubation Time

A549 cells were detached and counted as previously described. Three different curves were prepared: medium–high confluency, ranging from ~1.8 × 10^4^ to ~9 × 10^4^ cells/cm^2^; low–medium confluency, spanning ~1.8 × 10^3^ to ~1.6 × 10^4^ cells/cm^2^; very low confluency, ranging from ~3.5 × 10^2^ to ~1.8 × 10^3^ cells/cm^2^. Diluted cell samples were prepared in complete DMEM (1% FBS) and seeded in triplicate in a 48-well plate (#677 180, Greiner Bio-One, Milan, Italy). Following an ON incubation under standard conditions to ensure a firm attachment to the plate, the medium was gently removed, and 350 µL of resazurin WS was added to each well. A triplicate of resazurin WS only (Blank) was prepared. Cells were then incubated using standard conditions for 0.5, 1, 2, 3, and 4 h. At each incubation time point, metabolized resazurin WS was removed from the wells, and 100 µL was transferred to a 96-well plate for FI detection. FI was measured with the EnSpire Multimode Plate Reader at *λ*_Ex_ 545 nm and *λ*_Em_ 590 nm [17].

### 2.5. Limit of Blank, Limit of Detection, and Limit of Quantification

The Limit of Blank (LoB), Limit of Detection (LoD), and Limit of Quantification (LoQ) were calculated using the calibration curve method [18,19,20]. Estimations for LoB, LoD, and LoQ were based on data derived from the calibration curve “very low confluency” (3.5 × 10^2^–1.8 × 10^3^ cells/cm^2^) after a 4 h incubation. Subsequently, a validation of LoD and LoQ was conducted by analyzing 10 replicates of samples prepared in proximity to the previously estimated values (10 replicates in the same experiment) [17].

### 2.6. Repeatability, Reproducibility, and Measurement Uncertainty

A549 cells were detached and counted as previously described. Three different cell confluences were selected: ~9 × 10^4^ cells/cm^2^ for high confluency, ~4.5 × 10^4^ cells/cm^2^ for medium confluency, and ~9 × 10^3^ cells/cm^2^ for low confluency. Cells dilutions were appropriately prepared in complete DMEM (1% FBS) and seeded in triplicate in a 96-well, 48-well, and 24-well plate (TCP011024, Biofil, Pisa, Italy). Cells were cultured ON using standard cell culture conditions to allow them to be firmly attached to the bottom of the plate. After gently removing the medium from the wells, resazurin WS was added to each well (100 µL for the 96-well, 350 µL for the 48-well, and 600 µL for the 24-well plate) [17]. A triplicate of resazurin WS only (Blank) was prepared for each plate type. Cells were incubated using standard conditions for 1.5 h. Then, metabolized resazurin WS was removed from the wells, and 100 µL was transferred to a 96-well plate for FI detection. FI was measured with the EnSpire Multimode Plate Reader at *λ*_Ex_ 545 nm and *λ*_Em_ 590 nm. This experiment was performed 3 times on 3 different days (Exp 1, Exp 2, Exp 3) by the same operator. Repeatability, reproducibility, and measurement uncertainty (MU) were evaluated [17,21,22,23].

### 2.7. Fibrin Gel Scaffold Preparation (3D Cell Culture)

Fibrin gel scaffolds were prepared following a synthesis protocol optimized by our group [24]. More in detail, bovine fibrinogen (Fraction I, type I-S from bovine plasma, Sigma Aldrich, St. Louis, MO, USA) was dissolved for about 2 h at 37 °C in 0.9% sodium chloride (NaCl) to prepare a 5 mg/mL solution; bovine thrombin (Sigma Aldrich, St. Louis, MO, USA) was dissolved in milliQ-H_2_O at a final concentration of 100 U/mL; a 50 mM calcium chloride (CaCl_2_) (Sigma Aldrich, USA) solution was prepared in milliQ-H_2_O. After complete homogenization, each solution was sterilized using a 0.22 µm filter. Fibrinogen solution was further diluted in complete DMEM (10% FBS) to a final concentration of 2.5 mg/mL before use (fibrinogen WS). Thrombin WS was prepared by mixing 100 U/mL thrombin and 50 mM CaCl_2_ to achieve final concentrations of 12.5 U/mL and 25 mM, respectively.

Fibrin gel scaffolds were formed in a 48-well plate. In each well, two fibrin gel layers were prepared:

- Bottom layer: Fibrin gel without cells, which prevents cells from sinking to the bottom of the plate during gelling. In each well, 135 µL of fibrinogen WS were mixed with 15 µL of thrombin WS and incubated at 37 °C for about 30 min.
- Top layer: Fibrin gel with cells. A549 cell line was detached and counted as previously described. Cells were resuspended in fibrinogen WS at different selected cell concentrations. After the complete gelling of the bottom layer, in each well, 135 µL of fibrinogen WS-A549 were mixed with 15 µL of thrombin WS and incubated at 37 °C for 12 h. After the complete gelling of the top layer, 150 µL of complete DMEM (10% FBS) was added over the scaffolds to avoid dehydration and allow cell growth.

For both layers, the final concentrations of fibrinogen, thrombin, and CaCl_2_ were 2.25 mg/mL, 1.25 U/mL, and 2.5 mM, respectively.

Two calibration curves ranging from ~4.5 × 10^3^ to ~9 × 10^4^ cells/cm^2^ were used to identify the optimal incubation time and estimate repeatability, reproducibility, and MU as previously described for 2D culture. LoB, LoD, and LoQ were estimated starting from the 2D data and experimentally validated as indicated above for 2D culture.

### 2.8. Residual Resazurin on the Cell-Seeded Scaffold

Eight fibrin gel scaffolds were prepared in 48-well plates: 4 without cells (Blank: B1, B2, B3, and B4) and 4 with A549 cells at a density of 2 × 10^4^ cells/scaffold, around 1.3 × 10^5^ cell/cm^3^ (Sample: S1, S2, S3, and S4). Scaffolds were prepared as previously described. Culture media were changed every 3 days. Scaffolds were incubated with resazurin WS for 4 h, according to the following scheme:

- B1/S1: days 1, 4, 7, and 11 (4 times);
- B2/S2: days 4, 7, and 11 (3 times);
- B3/S3: days 7 and 11 (2 times);
- B4/S4: day 11 (1 time).

After each incubation, resazurin WS was removed from the well for FI measurement, and scaffolds were washed twice with 1× PBS before the addition of complete DMEM (10% FBS). Culture media were collected before each resazurin WS treatment, and FI was immediately (within 10 min time lapse) measured to evaluate the residual signal caused by the previous resazurin treatments. FI differences between samples treated 1, 2, 3, or 4 times with resazurin WS were evaluated to investigate any potential interference due to residual resazurin in the gel.

### 2.9. Optical Microscopy

Images of A549 cells, grown in fibrin gel and treated with resazurin, were acquired using a Zeiss Axio Observer. Z1/7 inverted microscope (Carl Zeiss, Oberkochen, Germany) with a 20× objective in phase contrast. Image analysis was conducted using Zeiss software ZEN 2.6 (blue edition).

### 2.10. Statistical Analysis

FI data were expressed as mean ± SD, where SD represents the standard deviation. Statistical analyses were performed using Origin 2022 software (OriginLab Corporation, Northampton, MA, USA). One-way ANOVA with post-hoc Tukey multiple comparisons was used to assess significant differences in FI values among sample groups. All analyses with a *p* < 0.05 were indicated as statistically significant (* *p* ≤ 0.05; ** *p* ≤ 0.01; *** *p* ≤ 0.001; **** *p* ≤ 0.0001).

## 3. Results and Discussion

### 3.1. Optimal λ_Ex_-λ_Em_ Wavelengths Identification

The optimal *λ*_Ex_-*λ*_Em_ combination for A549 cell lines treated with 44 µM Resazurin WS was determined by evaluating 12 different *λ*_Ex_-*λ*_Em_ combinations across 3 different cell confluences: low, medium, and high. The linearity of the results was evaluated for each *λ*_Ex_-*λ*_Em_ combination, all of which exhibited high linearity with coefficient of determination (R^2^) values ranging between 0.982 and 0.998.

**Figure 1:**
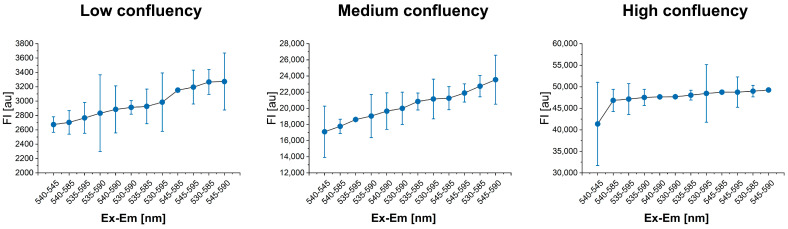
FI_Sample-Blank_ (*y*-axis) versus *λ*_Ex_-*λ*_Em_ conditions (*x*-axis) for low, medium, and high cell confluency. FI is expressed as arbitrary units (au). Error bars indicate SD.

To select the best *λ*_Ex_-*λ*_Em_, results were analyzed based on the cell confluency. No disagreements were observed for the high confluency (Figure 1). However, for both low and medium cell confluences, the *λ*_Ex_-*λ*_Em_ combination of 545 nm as *λ*_Ex_ and 590 nm as *λ*_Em_ yielded the greatest FI difference between the experimental well and the Blank (Figure 1). The *λ*_Ex_ of 545 nm and *λ*_Em_ of 595 nm were selected as optimal conditions for resazurin assay in A549 cell lines (R^2^ = 0.985), and this *λ*_Ex_-*λ*_Em_ combination was used for the subsequent experiments.

### 3.2. Optimal Incubation Time Evaluation

**Figure 2:**
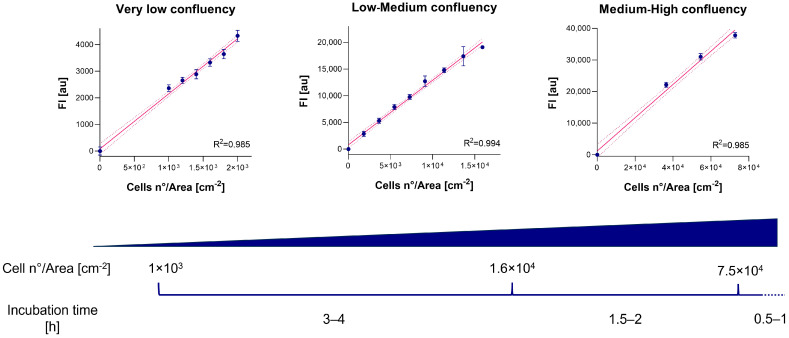
FI_Sample-Blank_ (*y*-axis) versus cell concentration (*x*-axis) for very low, low–medium, and medium–high cell confluency. FI is expressed as arbitrary units (au). Error bars indicate SD. Linearity was indicated by R^2^ values. Suggested optimal incubation time is indicated for different cell concentration ranges.

The selection of incubation time is strictly dependent on cell type and concentration. Optimal incubation times for A549 cells treated with 44 µM resazurin WS were identified by evaluating three different dilution curves at very low, low–medium, and medium–high confluency. After treating cells with resazurin WS, FI was measured at 0.5, 1, 2, 3, and 4 h of incubation under standard conditions. Results indicated that 3–4 h is the optimal incubation time when the cell concentration ranges from ~1 × 10^3^ to ~1.6 × 10^4^ cells/cm^2^; 1.5–2 h for cell concertation between ~1.6 × 10^4^ and ~7.5 × 10^4^ cells/cm^2^; 0.5–1 h for cell concertation exceeding ~7.5 × 10^4^ cells/cm^2^ (Figure 2). Prolonged incubation times, in relation to the number of cells, resulted in a plateau in the curve (Figure S1). This is due to the loss of direct correlation between the resazurin reduction and the number of viable cells.

### 3.3. Calculation of LoB, LoD, and LoQ

**Figure 3:**
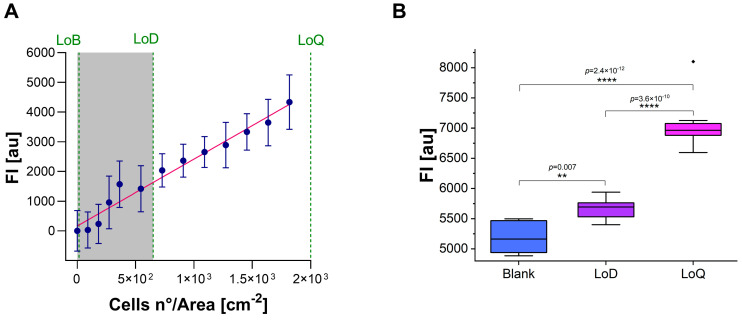
(**A**) LoB, LoD, and LoQ estimation by calibration curve method. FI_Sample-Blank_ (*y*-axis) versus cell concentration (*x*-axis). FI is expressed as arbitrary units (au). Error bars indicate SD. The green dotted lines indicate LoB, LoD, and LoQ; the gray area indicates the range of the curve in which linearity is lost. (**B**) Experimental validation of LoD and LoQ. FI is expressed as arbitrary units (au). Blank represents resazurin WS only. ** *p* ≤ 0.01; **** *p* ≤ 0.0001.

The LoB, LoD, and LoQ were estimated by using a calibration curve ranging from ~3.5 × 10^2^ to ~1.8 × 10^3^ cells/cm^2^ (very low confluency). Regression analysis was performed to evaluate the curve slope (S) and the SD of the y-intercept. LoB was estimated as ~15 cells/cm^2^, while LoD and LoQ were estimated to be approximately 6.5 × 10^2^ cells/cm^2^ and 2 × 10^3^ cells/cm^2^, respectively, for the resazurin assay on A549 cell lines (Figure 3A). Subsequent analysis confirmed the loss of curve linearity under the LoD (R^2^ = 0.80).

To experimentally validate the estimated values, 10 replicates of samples prepared with a concentration of 6.5 × 10^2^ cells/cm^2^ (LoD) and 2 × 10^3^ cells/cm^2^ (LoQ) were analyzed. Results showed a coefficient of variation (CV%) of 66.2% and 15.6% for LoD and LoQ, respectively. Although the FI value of both LoD and LoQ were significantly different from the Blank sample (LoD vs. Blank: *p* = 0.007; LoQ vs. Blank, *p* = 2.42 × 10^−12^), the CV% of LoD was too high to ensure reliable results, so the limit of ~2 × 10^3^ cells/cm^2^ (LoQ) was fixed as the minimum recommended cell concentration for proliferation/cytotoxicity test using resazurin assay on A549 cell line (Figure 3B).

### 3.4. Repeatability, Reproducibility, and Measurement Uncertainty

**Figure 4:**
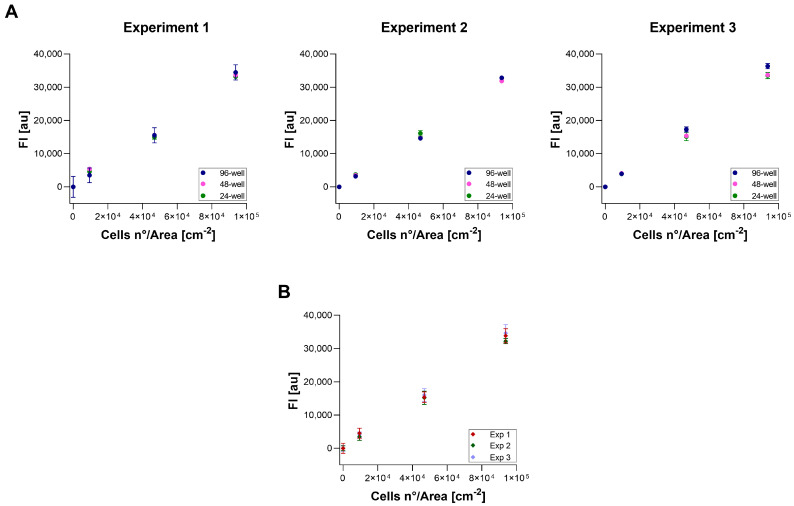
FI (*y*-axis) versus cell concentration (*x*-axis). FI is expressed as arbitrary units (au). Error bars indicate SD. (**A**) Differences between FI results obtained in 96-well, 48-well, and 24-well plates in all the experiments (Exp). (**B**) Comparison of FI results obtained for each confluency condition in each experiment (Exp).

Repeatability, reproducibility, and MU were calculated for three different cell confluences: low, medium, and high. The linearity of the curves was assessed, yielding an R^2^ mean of 0.994 ± 0.002 (mean ± SD). No significant differences were found between 96-well, 48-well, and 24-well plates (Figure 4A). Consequently, results from the different plates were considered together for repeatability, reproducibility, and MU calculations. Figure 4B shows the results obtained for each confluency in each experiment (*n* = 3). Our data indicated a mean relative repeatability of 4.5% (7.1% for Blank, 4.7% for low, 4% for medium, and 3.1% for high confluency) and a mean relative reproducibility of 5.5% (7.3% for Blank, 7% for low, 4.2% for medium, and 3.4% for high confluency). In each experiment, with a reading volume of 100 μL, the uncertainty due to pipetting error was 0.53%. The mean relative expanded uncertainty (U%) for resazurin assay on the A549 cell line was found to be optimal, measuring at 14.2% (20.3% for Blank, 16.9% for low, 11.6% for medium, and 8.1% for high confluency).

### 3.5. Resazurin Assay on 3D Cell Culture

**Figure 5:**
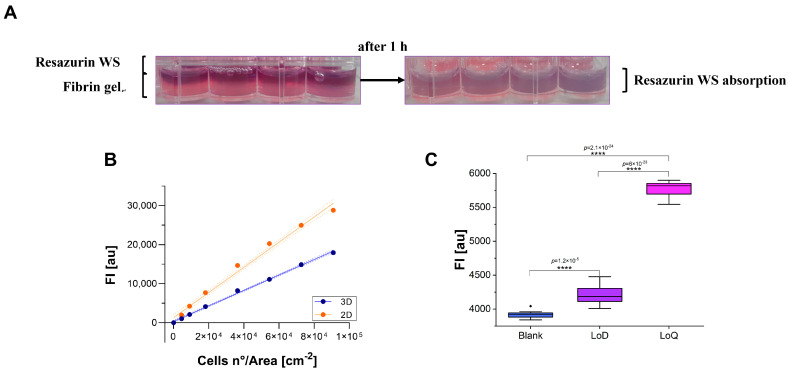
(**A**) Resazurin WS absorption by fibrin gel after 1 h of incubation. (**B**) Comparison between 2D (orange) and 3D (blue) identical curves incubated 1.5 h and 3 h, respectively. FI_Sample-Blank_ (*y*-axis) versus cell concentration (*x*-axis). FI is expressed as arbitrary units (au). Error bars indicate SD. (**C**) Experimental validation of LoD and LoQ in 3D culture model. FI is expressed as arbitrary units (au). Blank represents resazurin WS only. **** *p* ≤ 0.0001.

The feasibility of using the resazurin assay for 3D proliferation/cytotoxicity tests was evaluated using A549 cells encapsulated in a fibrin gel-based scaffold. First, we assessed the fibrin gel’s ability to absorb resazurin WS, observing complete absorption after 1 h of incubation (Figure 5A). Successively, we compared 2D and 3D A549 cell cultures. Two identical calibration curves were prepared in a 48-well plate for 2D and 3D by seeding or encapsulating A549 cells. The optimal incubation time for A549 in fibrin gel was found to be approximately double that of the time used for 2D culture. Under these conditions, curves exhibited R^2^ values of 0.997 and 0.988 for 3D and 2D culture models, respectively (Figure 5B).

Repeatability, reproducibility, and MU for the 3D culture model were calculated as previously described and were comparable with the 2D model. In detail, the results showed a mean relative repeatability of 4.3% and a mean relative reproducibility of 6.9%. The uncertainty due to the pipetting error of reading volume was the same as 2D. The mean relative expanded uncertainty (U, expressed as %) for resazurin assay on A549 cell lines inside fibrin gel 3D culture was found to be 16.5%.

LoD and LoQ, estimated starting from 2D data, were found to be ~8.8 × 10^3^ cells/cm^3^ and ~2.64 × 10^4^ cells/cm^3^, respectively. Experimental validation of these values, with 10 replicates of samples prepared at concentrations of 8.8 × 10^3^ cells/cm^3^ (LoD) and 2.64 × 10^4^ cells/cm^3^ (LoQ), indicated a CV% of 54.9% and 6.7% for LoD and LoQ, respectively. LoB was estimated as ~800 cells/cm^3^. Similar to the 2D culture model, although the FI value of both LoD and LoQ were significantly different from the Blank sample (LoD vs. Blank: *p* = 1.16 × 10^−5^; LoQ vs. Blank, *p* = 2.12 × 10^−24^), but the CV% associated with LoD was excessively high to ensure reliable results (Figure 5C). Therefore, the limit of 2.64 × 10^4^ cells/cm^3^ (LoQ) was established as the minimum recommended cell concentration for proliferation/cytotoxicity tests using resazurin assay on A549 cell lines in 3D (fibrin gel).

### 3.6. Residual Resazurin on the Fibrin Gel Scaffold

**Figure 6:**
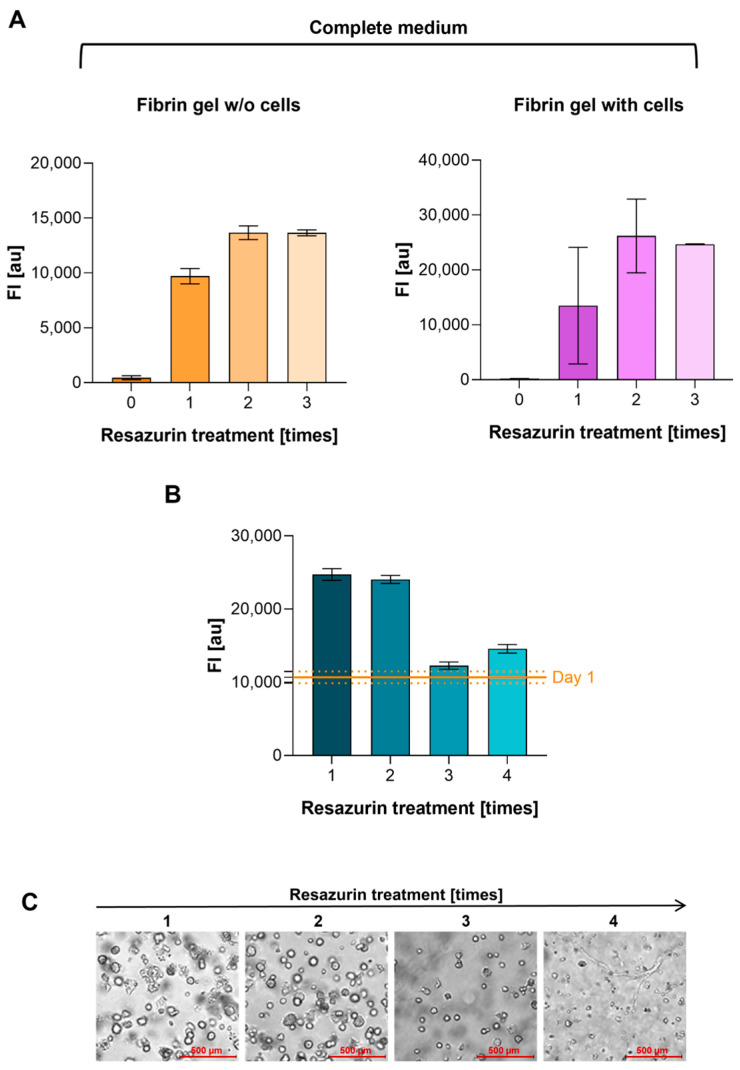
(**A**) FI values (*y*-axis) measured in cell culture media collected from fibrin gels without (w/o) and with cells before each resazurin WS treatment (*x*-axis). The complete medium was used as a control (resazurin treatment = 0). FI is expressed as arbitrary units (au). Error bars indicate SD. (**B**) FI_Sample-Blank_ results (*y*-axis) in identical samples treated 1, 2, 3, or 4 times with resazurin WS (*x*-axis) at day 11. The FI result of the day of seeding (day 1) are indicated by continuous orange line, while dashed lines indicate ± SD. FI is expressed as arbitrary units (au). Error bars indicate SD. (**C**) Images acquired with optical microscopy on day 11 of identical samples treated 1, 2, 3, or 4 times with resazurin WS (20× objective).

When 2D and 3D results were compared, a ratio between FI values (2D vs. 3D) of 1.81 ± 0.13 (mean ± SD) was observed. It is plausible to assume that part of the resazurin/resorufin remains trapped in the gel. To verify the possibility of measuring several times the same fibrin gel scaffold without any interference caused by resazurin/resorufin residual, four identical scaffolds containing the same concentration of A549 cells (S1, S2, S3, and S4) and four scaffolds without cells (B1, B2, B3, and B4) were prepared. S1 and B1 were treated with resazurin WS 4 times, S2 and B2 3 times, S3 and B3 twice, and S4 and B4 once. Culture media were collected from fibrin gels without cells (B1, B2, B3, and B4) and with cells (S1, S2, S3, and S4) before each resazurin WS treatment, and only the FI of the media was measured to evaluate the signal due to any residual resazurin in the gel due to previous resazurin treatments. The complete medium was used as a control (resazurin treatment = 0). It was observed that FI values significantly increased after one resazurin treatment, with a slight increment after 2 and 3 resazurin treatments, confirming that residual resazurin/resorufin remains trapped in the gel and is slowly released over the days following incubation (Figure 6A).

After 11 days of growth in the fibrin gels, we evaluated the FI results in the identical samples treated 1, 2, 3, or 4 times with resazurin WS. Considering that part of resazurin remains trapped in the gel, it was expected that FI values would be falsely higher in samples that underwent multiple measurements. Surprisingly, results indicated similar values in the samples that were treated only 1 or 2 times with resazurin (S4 and S3); on the contrary, samples S1 and S2, treated respectively 4 and 3 times with resazurin, showed FI values similar to those evaluated on the day of seeding (day 1) (Figure 6B).

To better understand the meaning of this result, cell morphology was analyzed with light microscopy. The images clearly showed that as the number of treatments with resazurin increases, the number of cells decreases, and their morphology worsens (Figure 6C). This underlines that the residual resazurin in the gel does not interfere with the FI measurement but is injurious to cells if subjected to long exposures. Therefore, our data suggest that performing time-lapse experiments involving more than two resazurin incubations is not advisable when cells are embedded in fibrin gel.

## 4. Conclusions

Given the crucial role A549 cells play in cytotoxicity studies and drug screening, the reliability of experimental outcomes becomes of primary importance. Utilizing A549 cells proves valuable in early drug discovery [5,25,26]; however, challenges in pre-clinical research, which were demonstrated by inconsistencies and reproducibility issues, need attention. The prevalence of data discrepancies in scientific studies, especially those involving cancer cell lines, underscores the requirement for standardized methodologies and transparent reporting [14,26].

Improving results reliability stands as a major challenge in the life sciences [27]. Regardless of the method employed to assess cellular responses to treatment, the obtained results should consistently align. Addressing these challenges not only contributes to the reliability of cytotoxicity results but also aligns with broader efforts within the scientific community to increase the consistency of pre-clinical research data [28,29].

To meet this challenge, an effective strategy should involve optimizing experimental parameters, specifically improving and standardizing laboratory protocols. In this manuscript, we applied and validated our previously described comprehensive optimization approach [17] to optimize the resazurin-based viability assay on the A549 cell line in both 2D and 3D (fibrin gel) in vitro models. This approach focused on critical experimental parameters and data quality assessment to support result robustness and reliability while characterizing and understanding the confidence limits of the test.

We successfully optimized the parameters for resazurin tests conducted on the A549 cell line in both 2D and fibrin gel 3D cultures and provided an SOP for conducting resazurin-based cytotoxicity assays specific for A549 cells in a 2D model, which ensures reliable results with minimal uncertainty of less than 15% (Supplementary File S1). This SOP can be useful in testing new biocompatible materials, performing drug screening, evaluating environmental toxins, and studying cellular responses to various treatments. It has a standard protocol comparable among different labs, thus ensuring data reliability and comparability.

While our optimized SOP offers enhanced reliability, it remains tailored to A549 cells. This approach can be adapted to the other adherent cell lines that are capable of metabolizing resazurin, but additional validation would be needed to confirm its effectiveness across various cell types and conditions.

Moreover, while the SOP was designed to standardize procedures across laboratories, some variability could still arise due to differences in reagents, equipment, and incubation conditions. A multi-laboratory validation would be beneficial to fully confirm the robustness of this protocol.

In conclusion, our optimized approach for resazurin-based assays on A549 cells provides a reliable tool for cytotoxicity testing, which advances the standardization necessary for robust pre-clinical drug screening.

## Data Availability

Data are contained within the article or supplementary material.

## Supplementary Materials

The following supporting information can be downloaded at https://www.mdpi.com/article/10.3390/cells13231959/s1, Supplementary File S1: Standardized protocol for resazurin-based viability assays on A549 cells in 2D culture; Supplementary Figure S1: Plateau curves for optimal incubation time variation. Refs [30,31] are citied in Supplementary Materials.
