# Supplementary material for: Standardized Protocol for Resazurin-Based Viability Assays on A549 Cell Line for Improving Cytotoxicity Data Reliability
Source: Cells. 2024 Nov 26;13(23):1959. doi: 10.3390/cells13231959 (PMC11640476; doi:10.3390/cells13231959)
Supplement: Supplementary file 1 [file cells-13-01959-s001.zip › Supplematry Figure S1.pdf]

Figure S1. Plateau curves for optimal incubation time variation.

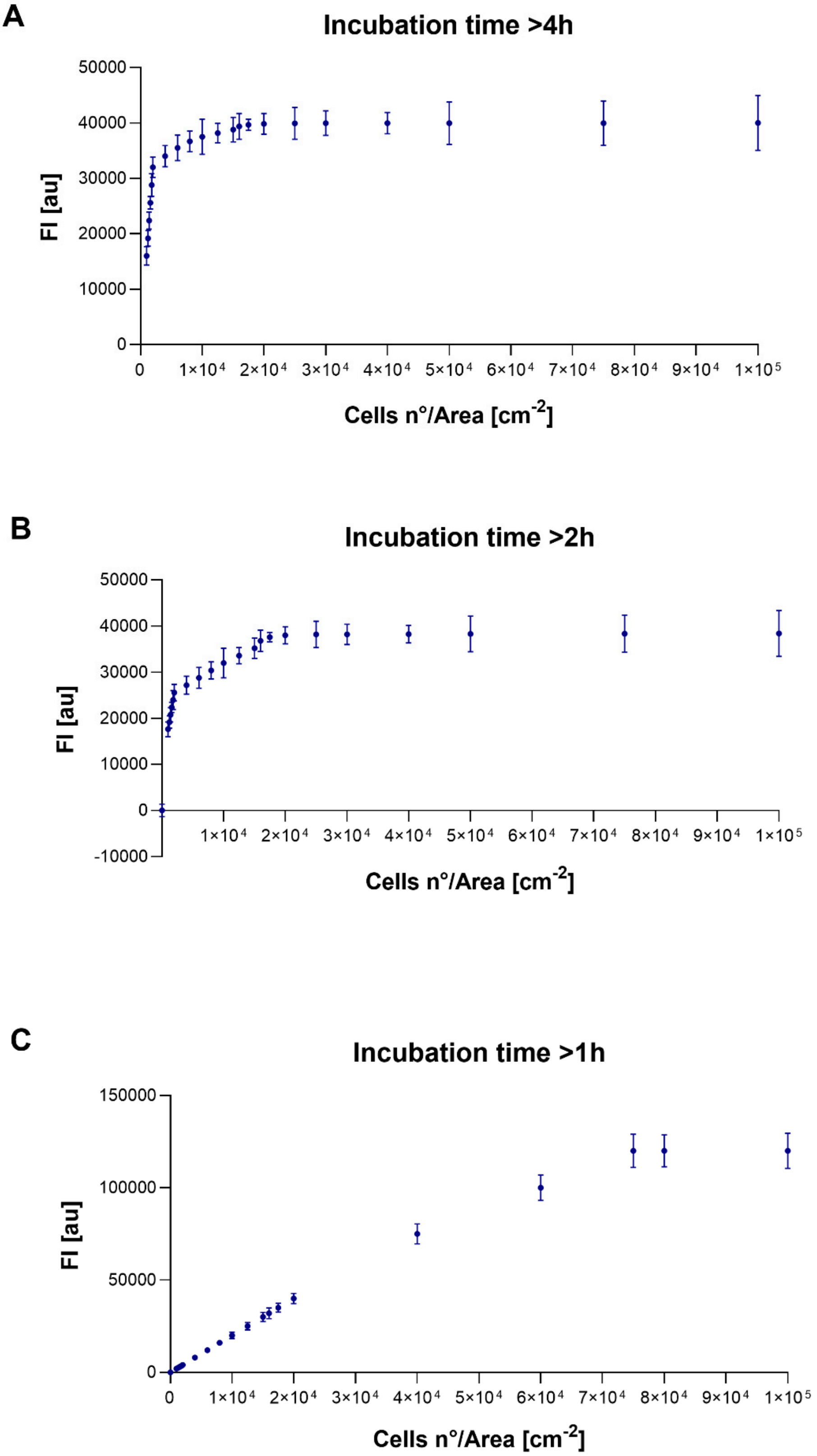

Figure S1. $FI_{Sample-Blank}$ (*y*-axis) versus cell concentration (*x*-axis) for all levels of confluency (very low, low-medium, and medium-high). FI is expressed as arbitrary units (au). Error bars indicate SD. Incubation time longer than **A)** 4 hours (h), **B)** 2h and **C)** 1h.
