# Supplementary material for: Standardized Protocol for Resazurin-Based Viability Assays on A549 Cell Line for Improving Cytotoxicity Data Reliability
Source: Cells. 2024 Nov 26;13(23):1959. doi: 10.3390/cells13231959 (PMC11640476; doi:10.3390/cells13231959)
Supplement: Supplementary file 1 [file cells-13-01959-s001.zip › Supplementary File S1.pdf]

SOP

Standardized protocol for resazurin-based viability assays on A549 cells in 2D culture

<table>
<tr><td colspan="3">Institution
Istituto Nazionale di Ricerca Metrologica – INRiM, Turin, Italy</td></tr>
<tr><td colspan="2">Title
Standardized protocol for resazurin-based viability assays on A549 cells in 2D culture</td><td>Date
2024-02-27</td></tr>
<tr><td>Document No.
INRIM_SOP Bio_002</td><td>First edition
2024-02-27</td><td>Version
1</td></tr>
<tr><td>Issued by
J. Petiti, S. Caria, M. Pegoraro</td><td>Reviewed by
L. Revel, C. Divieto</td><td>Approved by
C. Divieto</td></tr>
<tr><td colspan="3">Scope
Step-by-step protocol to assay viability on A549 cell line in 2D culture using a resazurin-based method with optimized parameters to ensure consistent results with a measurement uncertainty <15%.</td></tr>
</table>

Index

Introduction 3
Aims 3
Terminology 3
List of abbreviations 3
Materials 4
Equipment *4*
Consumables *4*
Reagents *4*
General information 4
Experimental workflow 5
Results quantification 7
Relative quantification *7*
Absolute quantification *7*
Test parameters 9
References 9

Introduction

The A549 cell line, derived from the lung tissue of a 58-year-old white male with lung cancer [1], has become a cornerstone in biomedical research, especially in respiratory and cancer studies. These cells exhibit unique properties that make them well-suited for investigations in cell biology, drug screening, and toxicity testing. Notably, the A549 cell line, that have been used for decades in thousands of studies, particularly as lung cancer model, play a pivotal role in cytotoxicity studies and drug screening, crucial for evaluating the impact of pharmaceutical compounds on cellular viability [2].

The resazurin-based viability assay is one of the most widely adopted tools for assessing drug cytotoxicity [3]. By harnessing the ability of living cells to reduce the non-fluorescent dye resazurin into fluorescent resorufin, this assay provides a reliable indicator of cellular metabolic activity and, consequently, cell viability. Despite its widespread use, the reliability of results from the resazurin-based viability test can be influenced by various factors, including cellular metabolic characteristics, environmental conditions, and experimental variables. The challenge of ensuring result consistency in pre-clinical drug screening, underscored by recently reported data inconsistencies [4–8], highlights the importance of using standardized protocols.

Aims

The primary objective of this Standard Operating Procedure (SOP) is to enhance the results reliability of the cytotoxicity test conducted on the A549 cell line using the resazurin-based assay. To address this aim, the SOP outlines a step-by-step optimized procedure for performing a viability assessment (cytotoxicity or proliferative test) with the resazurin assay on A549 cells. The adherence to this SOP ensures to obtain consistent results with a measurement uncertainty less than 15%.

Terminology

In the following SOP, the term “must” is used to denote mandatory steps or requirements within the protocol. When "must" is employed, adherence is non-negotiable, as these actions are essential for the correct execution and reliability of the procedure. Conversely, the term "should" has been employed to highlight recommended practices that, while not mandatory, are strongly advised for achieving optimal results.

List of abbreviations

FC, Fold Change; FBS, Fetal Bovine Serum; FI, Fluorescent Intensity; h, Hours; λ_{Em}, Emission Wavelength; λ_{Ex}, Excitation Wavelength; PBS, Phosphate Buffered Saline; RT, Room Temperature; SD, Standard Deviation; SOP, Standard Operating Procedure; WS, Working Solution.

Materials

Equipment

- Standard equipment for cell culture (e.g., centrifuge for tubes, cell incubator, laminar flow hood, optical microscope, micropipettes, etc.);
- Cell counting chamber or automatic cell counter;
- Fluorescence reader with monochromator or suitable wavelength filters.

Consumables

- Cell culture plates;
- 96-well plate for fluorescence measurements (e.g., black plates);
- Centrifuge tubes (15 and 50 mL);
- Microtubes (1.5 and 2 mL);
- Pipette tips;
- Serological Pipettes;
- 0.22 µm filter;
- Syringes.

Reagents

- Cell culture medium (F-12K medium or DMEM);
- Cell culture medium supplements to support cell growth and viability: fetal bovine serum (FBS), L-Glutamine (if not contained in the medium), optional antibiotic (e.g., penicillin/streptomycin or gentamicin);
- Trypsin-EDTA or equivalent;
- milliQ-H_2O;
- Resazurin sodium salt;
- Phosphate Buffered Saline (PBS).

General information

Unless stated otherwise, all procedures should be performed under sterile laminar flow conditions. Protective gloves and lab coat should be worn, and all work areas should be thoroughly cleaned before and after testing.

Experimental workflow

1. Dissolve resazurin powder into Mill-Q H_2O or 1x PBS to an appropriate concentration (e.g., 4.4 mM or 440 µM), sterilize by filtration through a 0.22 µm filter, and prepare aliquots to store at -20°C;
2. Prepare complete medium: F-12K medium or DMEM plus 10% FBS, 2 mM Glutamine, and 1% penicillin/streptomycin (optional);
3. Remove and discard exhaust culture medium;
4. Briefly rinse the cell layer with 1x PBS to remove all traces of FBS that contains trypsin inhibitors;
5. Add Trypsin-EDTA (or equivalent) solution[1] to the plate and put the cells in the incubator for 5 minutes (min)[2];
6. Add complete growth medium (approximately twice the volume of Trypsin-EDTA used), aspirate cells by gently pipetting, and transfer in a conical tube;
7. Centrifuge cells at 125g for 5 min;
8. Resuspend cells in an appropriate volume of medium and count them;
9. Seed an adequate number of cells in the multi-well plate chosen for the experiment. 70-80% cell confluency[3] ($5\text{-}6\times10^4$ cells/cm^2) is recommended to test compounds cytotoxicity; while 15-30% cell confluency ($1\text{-}2\times10^4$ cells/cm^2) is suggested for proliferation assays;
10. Include the following control samples:
    a. Untreated Cells Control: wells with untreated cells to serve as a "starting point", for cell proliferation experiments, or as a "100% living cell point", for cytotoxicity tests. Add the same solvent used to deliver the test compounds;
    b. Positive Control: wells containing cells treated with a known cell-proliferation-inducing compound for cell growth tests or a highly toxic substance (e.g., 15% DMSO) in cytotoxicity tests;
11. Culture cells until they are firmly attached to the bottom of the plate using standard cell culture conditions (recommended time: 4-6 hours (h));
12. Gently remove the complete medium from the well and treat cells with the test compound (e.g., growth factors, cytokines, or cytotoxic compounds) for a period of time that depends on your experiments (usually, 24-72 h);
13. After culturing cells for the desired exposure period, allow resazurin to reach room temperature (RT). A 37°C water bath may be used to eventually thaw the reagent. Protect the resazurin from direct light;
14. Prepare the resazurin working solution (WS) with a concentration of 44 µM in the complete cells culture medium and warm it at 37°C until use;
15. Remove the assay plate from the incubator and gently eliminate the medium from the wells;

[1] Volume depends on the plate surface. Refer to the manufacturer's recommendation to select the appropriate volume of Trypsin-EDTA (or equivalent) solution to add to your plate.
[2] To avoid clumping do not agitate the cells by hitting or shaking the flask while waiting for the cells to detach.
[3] Cell confluency is the degree to which the surface of a cell-culture dish is covered by adherent cells [9].

16. Add an appropriate volume of resazurin WS in each well (test and control wells). The volume of resazurin WS depends on the multi-well plate used. Refer to the table below to select the correct resazurin WS volume[4] (Table S1)

Culture plate	Surface area [cm^2]	Resazurin WS volume [mL]
96-well	0.32	0.1
48-well	1.1	0.35
24-well	1.9	0.6
12-well	3.5	1.1
6-well	9.6	3

Table S1. Resazurin WS volumes recommended for different multi-well plates in order to maintain the scalability ratio.

17. The following additional control samples are recommended in each experiment:
    a. No-Cell Control (Blank): resazurin WS only (wells without cells to serve as the negative control to determine fluorescent intensity (FI) background);
    b. Test Compound Control: wells without cells containing the solvent and compound to test for possible chemical interference with resazurin;
    c. Empty wells (two or more) in the 96-well plate for FI detection: empty wells should display a minimal FI signal. This control sample is useful to verify that the plate reader is working correctly[5].
18. Incubate cells using standard culture conditions. To select the optimal incubation time, refer to the following recommendations (Table S2):

Cell n°/Area [cm^{-2}]	Incubation time [h]
1×10^3 -1.6×10^4	3-4
1.6×10^4 -7.5×10^4	1.5-2
>7.5×10^4	0.5-1

Table S2. Resazurin incubation time recommended for different cell concentrations.[6]

19. Reached the expected incubation time, gently remove the resazurin WS from the respective wells and transfer 100 µL in a 96-well plate for FI measurement;
20. Use a Fluorescence Microplate Reader to record the FI at an excitation wavelength (λ_{Ex}) of 545 nm and an emission wavelength (λ_{Em}) of 590 nm;
21. Calculate the FI_{mean} and standard deviation (*SD*) of replicates for each test condition (test and controls wells);

[4] In line with the recommended volume of 100 µL/well for the 96-well plate, the volumes suggested for the other plates are proportionally adjusted based on their well surface area. This ensures that, regardless of the surface, cells may receive and metabolize an equivalent amount of Resazurin. Consequently, this approach guarantees the comparability of results obtained across different plates.
[5] This control sample is optional, but recommended at least in the first experiments.
[6] The incubation time is inversely proportional to the number of cells.

22. Subtract the FI_{mean} of Blank from the FI_{mean} of all experimental wells ($FI_{Sample\text{-}Blank}$);
23. Calculate the *SD* ($FI_{Sample\text{-}Blank}$) by propagating the error using the formula:

$$\boldsymbol{SD(FI_{Sample-Blank}) = \sqrt{SD^2_{Sample} + SD^2_{Blank}}}$$

Results quantification

Relative quantification

For proliferation assays, results are usually expressed as fold change (FC, a measure that describes how much a quantity changes between an original and a subsequent measurement [9]), normalizing results on "Untreated Cells Control" using the equation:

$$\boldsymbol{Cell\ growth_{FC} = \frac{FI_{Compound}}{FI_{Untreat\ Cells\ Control}}}$$

$FI_{Compound}$ = FI values of cells treated with different concentrations of compound
$FI_{Untreated\ Cells\ Control}$ = FI value of Untreated Cells Control

For cytotoxicity tests, results are usually expressed as percentage, normalizing on control samples by setting the Untreated Cells Control as "100% living cell point" and the Positive Control as "0% living cell point", applying the following formula ("min-max normalization" method, also called "feature scaling"):

$$\boldsymbol{z_s = \left(\frac{x_{s-\min(x)}}{\max(x) - \min(x)}\right) \times 100}$$

z_s = normalized value of the sample in the dataset
x_s = value of the sample in the dataset
min(x) = the minimum value in the dataset (Positive Control)
max(x) = the maximum value in the dataset (Untreated Cells Control)

Absolute quantification

Calibration curve preparation:

1. In a range between 4 and 6 h before the conclusion of the experiment, detach cells as indicated above;
2. Resuspend cells in an appropriate volume of medium and count them;
3. Prepare at least 5 cell serial dilutions in a complete culture medium. The standard concentrations should at least cover the range of estimated concentrations for the unknown test samples and be evenly spaced throughout the range[7] [10]. Use a cell culture medium with low FBS concentration (e.g., 1-2% FBS) to prevent excessive cell growth;
4. Seed the cell dilutions in a multi-well plate (at least triplicate wells for each condition are recommended);

[7] For a more convenient estimation of the calibration curve range, consult literature data, focusing on specific parameters relevant to the experiment type (e.g., cell duplication time under normal conditions, IC50, or LD50 of the tested compound).

5. Culture cells until they are firmly attached to the bottom of the plate using standard cell culture conditions.

Cell concentration evaluation:

6. Simultaneously with the addition of resazurin WS to the experiment samples, treat the calibration curve equally;
7. Reached the expected incubation time, gently remove the resazurin WS from the wells and transfer 100 µL in a 96-well plate for FI measurement;
8. Use a Fluorescence Microplate Reader to record the FI at λ_{Ex}: 545 nm and λ_{Em}: 590 nm;
9. Calculate the FI_{mean} and *SD* of replicate for each test condition (calibration curve, unknown samples, and controls);
10. Subtract the FI_{mean} of Blank from the FI_{mean} of all experimental wells and calibration curve ($FI_{Sample\text{-}Blank}$);
11. Calculate the *SD* ($FI_{Sample\text{-}Blank}$) by propagating the error using the formula:

$$\boldsymbol{SD(FI_{Sample-Blank}) = \sqrt{SD^2_{Sample} + SD^2_{Blank}}}$$

12. For the calibration curve, plot $FI_{Sample\text{-}Blank}$ (*y*-axis) versus the number of cells seeded (*x*-axis; e.g., cell n°/well or cell n°/cm^2)**;**
13. Calculate the linear equation resulting from the calibration curve. Check that R^2 is higher than 0.97. If R^2 is lower, repeat the experiment or exclude one or more points of the curve, avoiding having a calibration curve with less than 5 points;
14. Use the resulting linear equation to estimate the cell concentration of unknown samples by interpolating their $FI_{Sample\text{-}Blank}$ values (*y* values) as follow:

$$\boldsymbol{x = \frac{y - b}{m}}$$

x: cell concentration of the unknown sample (unknown variable)
y: $FI_{Sample\text{-}Blank}$ of the unknown sample
b: *y*-intercept of the linear equation derived from the calibration curve
m: slope (*S*) of the linear equation derived from the calibration curve

15. If the cell concentration values obtained for unknown samples were outside the calibration curve range, it is recommended to repeat the experiment by varying the calibration curve range.

N.B.: A calibration curve must be used in each independent experiment and must be incubated with resazurin WS under identical conditions as the unknown and control samples.

Test parameters

Test parameters	Values
Limit of Blank (LoB)	~15 cell/cm^2
Limit of Detection (LoD)	~6.5×10^2 cell/cm^2
Limit of Quantification (LoQ)	~2×10^3 cell/cm^2
Relative Repeatability	4.5%
Relative Reproducibility	5.5%
Relative Expanded Uncertainty	14.2%
Suitable for time lapse experiments	Yes

Table S3. Test parameters[8] associated with the specific protocol outlined in this SOP

References

1. Lieber M, Todaro G, Smith B, Szakal A, Nelson-Rees W. A continuous tumor-cell line from a human lung carcinoma with properties of type II alveolar epithelial cells. Intl Journal of Cancer [Internet] 1976 [citato 2023 nov 13];17:62–70. Available from: https://onlinelibrary.wiley.com/doi/10.1002/ijc.2910170110

2. Garcia-de-Alba C. Repurposing A549 Adenocarcinoma Cells: New Options for Drug Discovery. Am J Respir Cell Mol Biol [Internet] 2021 [citato 2023 nov 13];64:405–6. Available from: https://www.atsjournals.org/doi/10.1165/rcmb.2021-0048ED

3. Rampersad SN. Multiple Applications of Alamar Blue as an Indicator of Metabolic Function and Cellular Health in Cell Viability Bioassays. Sensors [Internet] 2012 [citato 2024 lug 5];12:12347–60. Available from: https://www.mdpi.com/1424-8220/12/9/12347

4. Hirsch C, Schildknecht S. In Vitro Research Reproducibility: Keeping Up High Standards. Front. Pharmacol. [Internet] 2019 [citato 2023 nov 14];10:1484. Available from: https://www.frontiersin.org/article/10.3389/fphar.2019.01484/full

5. Reynolds PS. Between two stools: preclinical research, reproducibility, and statistical design of experiments. BMC Res Notes [Internet] 2022 [citato 2023 nov 14];15:73. Available from: https://bmcresnotes.biomedcentral.com/articles/10.1186/s13104-022-05965-w

6. Baker M. 1,500 scientists lift the lid on reproducibility. Nature [Internet] 2016 [citato 2023 nov 14];533:452–4. Available from: https://www.nature.com/articles/533452a

7. Haibe-Kains B, El-Hachem N, Birkbak NJ, Jin AC, Beck AH, Aerts HJWL, et al. Inconsistency in large pharmacogenomic studies. Nature [Internet] 2013 [citato 2023 mag 10];504:389–93. Available from: https://www.nature.com/articles/nature12831

8. Freedman LP, Cockburn IM, Simcoe TS. The Economics of Reproducibility in Preclinical Research. PLoS Biol 2015;13:e1002165.

9. Borowski EJ, Borwein JM. Collins dictionary of Mathematics. In: Collins dictionary of Mathematics. Harper Collins; 2012.

10. Barwick V. Preparation of Calibration Curves - A Guide to Best Practice [Internet]. 2003;Available from: https://www.lgcgroup.com/media/2997/preparation-of-calibration-curves.pdf?ipignore=true

[8] Refer to the main text for parameter definitions and calculation.
